# Shifting Attitudes from Willingness to Uptake in COVID-19 and Influenza Vaccination—Associated Factors and Reported Reasons

**DOI:** 10.3390/vaccines14070555

**Published:** 2026-06-25

**Authors:** Sara Moura, António Teixeira Rodrigues, Sónia Romano, Nuno Rodrigues, José Guerreiro, Ema Paulino, André Peralta-Santos

**Affiliations:** 1 Oeste Local Health Unit, Torres Vedras, Portugal; sara.moura@ulso.min-saude.pt (S.M.); nuno.s.rodrigues@ulso.min-saude.pt (N.R.); 2 NOVA National School of Public Health, NOVA University Lisbon, Lisbon, Portugal; 3 Cientis, Lisbon, Portugal; sonia.romano@anf.pt (S.R.);; 4 National Association of Pharmacies (ANF), Lisbon, Portugal; 5 Life and Health Sciences Research Institute (ICVS), School of Medicine, University of Minho, Braga, Portugal; 6 ICVS/3B’s-PT Government Associate Laboratory, Braga/Guimarães, Portugal; 7 Comprehensive Health Research Center, CHRC, NOVA University Lisbon, Lisbon, Portugal; 8 Direção-Geral da Saúde, Alameda D. Afonso Henriques, 45, 1049-005 Lisbon, Portugal

**Keywords:** hesitancy, refusal, influenza, COVID-19, vaccine

## Abstract

Background/Objectives: Vaccine hesitancy is a complex and growing phenomenon worldwide, posing a serious threat to public health achievement in disease control and prevention. This study aimed to assess willingness to uptake and factors linked to shifts between different categories of willingness and uptake regarding the COVID-19 and influenza vaccines. Methods: Prospective cohort study with a representative sample of 1400 individuals aged ≥60 years residing in mainland Portugal, randomly selected. Two telephone surveys were conducted: one at the start of the 2023/2024 vaccination campaign, assessing patients’ characteristics and willingness for vaccination (using an 11-point Likert scale), and another at the end, assessing vaccination status and reasons for uptake/non-uptake. Results: Shifts were observed among both acceptance and refusal groups—12.93% of the individuals within these categories shifted to an opposite decision. Hesitancy presents divergent attitudes: for the COVID-19 vaccine, 56.50% declined vaccination, while for the influenza vaccine, non-uptake was only 30.60%. Age, presence of chronic disease, level of education, household dimension, and previous uptake of booster doses are significantly associated with shifting attitudes, playing different roles for each category of willingness and uptake outcome. For the acceptance category, non-uptake relates to confidence factors. For hesitancy, non-uptake is mainly due to complacency. For refusal, the decision is influenced by all domains. Conclusions: Vaccine hesitancy remains an important public health concern in the Portuguese population and appears to differ between COVID-19 and influenza vaccination. Attitudes toward COVID-19 and influenza vaccines can vary in all directions over a short period. Acceptance does not guarantee uptake, and refusal can shift towards uptake. These findings highlight the importance of reinforcing public health strategies and interventions for uptake across a population, taking into consideration the specificities of each willingness group.

## 1. Introduction

Vaccination is one of the most effective and safe public health strategies for disease prevention [1]. Vaccine hesitancy, defined by the World Health Organization (WHO) as the “delay in acceptance or refusal of vaccines despite availability of vaccination services” ref [2], represents a serious threat to the progress achieved through vaccination. This is a complex phenomenon, as even vaccinated individuals may harbor doubts or reluctance towards vaccination. It has an individual character, influenced by a wide range of factors, such as past experiences or knowledge about the vaccine. However, it is also shaped by the context in which the individual is embedded and must always be analyzed within the historical, sociocultural, and political context [3].

According to the Strategic Advisory Group of Experts on Immunization Working Group on Vaccine Hesitancy (SAGE), the factors contributing to hesitancy can be explained by the “3Cs” model: complacency, confidence, and convenience. Complacency refers to the perception that the disease targeted by vaccination does not pose a significant threat. Confidence concerns trust in vaccines, healthcare professionals, and the institutions promoting them. Convenience relates to the ease of access to vaccination, with logistical barriers such as costs, inconvenient schedules, and distance reducing vaccine uptake [2].

For both COVID-19 and influenza, some causes of vaccine hesitancy are shared across countries (such as safety concerns), but others are country- and culture-related. Thus, enhancing country-specific and group-specific knowledge is crucial to allow targeted interventions from accredited sources [4].

A study conducted in Portugal highlighted greater COVID-19 vaccine hesitancy among younger age groups, suggesting this may be a growing phenomenon in the country. The same study concluded that the number of hesitant individuals and the clusters identified are concerning in a country historically highly receptive to vaccination [5]. In Portugal, the main reasons reported for non-vaccination against influenza among individuals aged 65 years and older include access barriers, lack of awareness of vaccination recommendations, distrust in the vaccine, and low perceived vulnerability [6].

Although vaccine hesitancy critically limits the achievement of desired coverage, it is not a static condition, as the proportion of individuals intending to vaccinate may vary substantially, even over short periods of time [7]. Given its complexity, hesitancy should not be assessed exclusively through coverage rates, as some hesitant individuals still get vaccinated, while others may be willing but face logistical limitations [2].

Some authors define vaccine hesitancy as “a state of indecisiveness regarding a vaccination decision,” referring not to a behavior, but to the decision-making process [8].

Even though several factors and reasons associated with hesitancy are documented, they should be consistently studied considering temporal, geographical, demographic, and vaccine-specific aspects. Furthermore, hesitancy must be systematically monitored and evaluated as a dynamic phenomenon, which, despite being acknowledged as such, still lacks studies that characterize the determinants of willingness and uptake mismatches [7].

The seasonal vaccination campaign aims to administer a booster dose of the COVID-19 vaccine and the influenza vaccine, both free of charge, to the same age category and under identical logistics conditions. In 2023/2024, in Portugal, significant changes were introduced, including an expansion of eligibility criteria for individuals aged over 65 to over 60 years old and an increase in vaccination sites by including community pharmacies, aiming to increase convenience, access, and, therefore, coverage [9,10].

This study aims to assess willingness to vaccination uptake and factors linked to shifts between different categories of willingness and uptake regarding the COVID-19 and influenza vaccines. Additionally, it aims to describe reasons for uptake/non-uptake within each shifting subgroup. Advancing knowledge on this subject enables public health to tailor more efficient interventions for disease prevention and control through vaccination. Moreover, it enables improved design of vaccination campaigns.

## 2. Materials and Methods

### 2.1. Study Design, Participants, and Variables

A prospective cohort study was conducted using telephone surveys (Computer-Assisted Telephone Interviewing, CATI) with a representative sample of 1400 individuals aged ≥60 years residing in mainland Portugal. Participants were randomly selected (stratified) from a list of national landline and mobile numbers accessed by an external subcontracted company.

Data collection took place in two waves: the first (T0), a baseline questionnaire (Q0), was administered before the start of the vaccination season (22–29 September 2023); and the second (T1), at the end of the seasonal vaccination campaign (25 January–7 February 2024), using a follow-up questionnaire (Q1). All participants included in the study (individuals who agreed to participate and gave oral informed consent) were required to take part in both data collection points. Anonymized data was shared with the research team.

Q0 included sociodemographic variables (age, sex, education level, and household size), clinical information (self-reported chronic disease and prior SARS-CoV-2 infection), previous vaccination status, and willingness for uptake during the 2023/2024 season. Participants were asked on an 11-point Likert scale from 0 (strongly disagree) to 10 strongly agree) to indicate their level of agreement with the following statement: “I intend to get vaccinated against influenza/COVID-19 in this upcoming seasonal vaccination campaign.” Responses were subsequently categorized as refusal for scores between 0 and 2, hesitancy for scores between 3 and 7, and acceptance for scores between 8 and 10 [11]. The selection of this instrument was motivated by its ease of application and the opportunity to compare findings with previous results obtained for the same vaccine within the Portuguese population. Sensitivity, specificity, positive predictive value (PPV), and negative predictive value (NPV) were tested within the realm of this study for all possible categorizations into three states (refusal, hesitancy, and acceptance). The selected model demonstrated adequate discriminative performance. For the influenza vaccine model, sensitivity was 0.74, specificity 0.69, positive predictive value (PPV) 0.83, and negative predictive value (NPV) 0.56. For the COVID-19 vaccine model, sensitivity was 0.74, specificity 0.57, PPV 0.66, and NPV 0.70. These values compared favorably with alternative models tested across different cut-off combinations.

Q1 collected information on self-reported vaccination status for influenza and COVID-19 in the 2023/2024 campaign, as well as reasons for uptake/non-uptake listed based on the previous literature review and international instruments (one or more reasons could be reported).

For this study purpose, hesitancy—as defined by the WHO—is divided into refusal (as an individual’s unavailability to uptake) and hesitancy (as a state of uncertainty toward uptake), aiming for a better understanding of different subgroups’ behavior.

### 2.2. Sample Size and Statistical Analysis

Based on prior experience with similar projects, an estimated dropout rate of approximately ±15% between questionnaires was anticipated. To ensure a final sample size of 600 individuals per target group (60–64 years and ≥65 years, representing the groups for whom vaccines were administered free of charge for the first time as well as the group eligible in previous campaigns) at T1, for a ±4.00% margin of error at a 95% confidence level, a total of 1400 individuals were recruited at T0 (700 per group), corresponding to a ±3.70% margin of error at the same confidence level. For estimates concerning the population aged 60 and over, sample data was weighted to adjust the results according to the age distribution of the Portuguese population (weight 0.44 for ages 60–64 and 1.56 for ≥65). The Chi-square test was used to compare patients who completed the follow-up and those who were lost to follow-up with respect to baseline characteristics (age, sex, education level, chronic disease, household, previous COVID-19 infection, and previous influenza vaccine uptake).

Intention and uptake estimates were computed with the corresponding 95% CIs. Data from individuals who exhibited different attitudes at the two time points during the campaign (T0 and T1) was analyzed. This variation was defined as a shift in attitude (the outcome of the analysis). A shift was defined as a situation when an individual moved from refusal to uptake, from acceptance to non-uptake, and from hesitancy to uptake or to non-uptake. For this analysis, the Chi-square test or Fisher’s exact test was applied.

Logistic regression models were used to identify factors associated with vaccine uptake or non-uptake within each baseline willingness category. For participants who initially reported acceptance, the outcome was shifting to non-uptake, with sustained uptake as the reference category. For participants who initially reported refusal, the outcome was shifting to uptake, with sustained non-uptake as the reference category. For participants who initially reported hesitancy, the outcome was uptake versus non-uptake at the end of the campaign. The exposure variables were selected a priori based on the previous literature and conceptual relevance to vaccine hesitancy and uptake, and included age, sex, education level, household size, chronic disease, prior SARS-CoV-2 infection for COVID-19-related models, and previous influenza vaccination for influenza-related models. The odds ratio (OR) and Wald’s 95% CIs were estimated. A full model was adopted, i.e., all variables were considered in the analysis. Model diagnoses included calculation of Hosmer and Lemeshow goodness-of-fit and the Variance Inflation Factor (VIF) to check for multicollinearity.

Reported reasons for the decision of uptake or non-uptake were presented as absolute (n) and relative (%) frequencies within each subgroup of shifting.

Statistical significance was set at 5%. Analysis was performed using SAS Enterprise Guide^®^ v7.15 software.

### 2.3. Ethical Considerations

The study was conducted in accordance with the principles of the Declaration of Helsinki. The research team consulted the responsible data protection officers and confirmed that all applicable General Data Protection Regulation (GDPR) requirements were met prior to and throughout data collection. The study protocol was approved by the Oeste Local Health Unit Ethics Committee (protocol code 33/CES/2025, 22 August 2025). All participants were informed regarding the study objectives and provided verbal informed consent before data collection. Researchers only had access to anonymized data.

## 3. Results

A total of 1400 valid responses were obtained in T0, and 1200 in T1. The response rate in T1 was 85.71% of the participants who responded to T0. A higher proportion of women (57.83% vs. 50.07%), patients with basic education (72.55% vs. 57.94%), and patients without previous COVID-19 infection (66.77% vs. 57.75%) was found in those who were lost to follow-up (*p* < 0.05).

A representative sample for the target population of 60 years old and over was obtained: 78.00% were aged 65 or over, and 22.00% aged 60–64; 51.19% were female; 57.99% had a basic education level, and 11.31% an advanced education level; 13.26% were living alone; and 44.98% had chronic disease.

### 3.1. Willingness and Shifting Towards Uptake/Non-Uptake

Most individuals showed acceptance for both vaccines before the beginning of the campaign. The influenza vaccine was slightly better accepted (72.96%, 95% CI 70.32–75.60) than the COVID-19 vaccine (68.32%, 95% CI 65.40–71.25). Characteristics of the population within each category of willingness to vaccinate for both COVID-19 and influenza are detailed in the Supplementary Materials Table S1.

**Figure 1:**
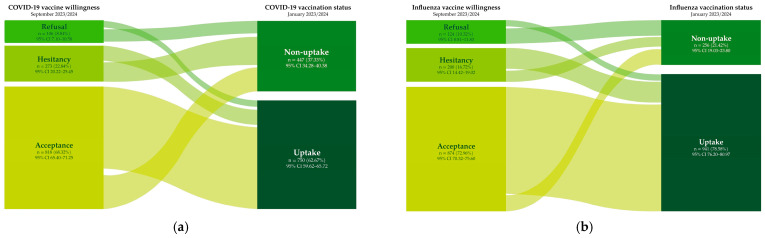
Distribution of the population by category of willingness to uptake (refusal, hesitancy, and acceptance) before the beginning of the 2023/2024 campaign (start point, September 2023) and their pathway to uptake or non-uptake at the end of the campaign (end point, February 2024), for the (**a**) COVID-19 vaccine and (**b**) influenza vaccine.

Different patterns of behaviors were observed among individuals showing any form of hesitancy: refusal was more common for the influenza vaccine, while hesitancy was more frequent for the COVID-19 vaccine. Detailed values are presented in Figure 1.

Overall, shifting was found to be possible for the acceptance and refusal groups, for both vaccines: 12.93% of the individuals within these categories shifted to an opposite decision by the end of the campaign. Individuals presenting hesitancy had diverging attitudes toward uptake for each vaccine. For the COVID-19 vaccine, the majority (56.50%) declined vaccination, contrary to the influenza vaccine, which presented 30.60% non-uptake among hesitant individuals (Table 1).

Refusal was found to be associated with a higher odds of non-uptake (influenza: OR 6.42, 95% CI 3.34–12.34; COVID-19: OR 6.12, 95% CI 3.53–10.60), and hesitancy was associated with a higher odds of non-uptake for the COVID-19 vaccine, but not for the influenza vaccine (COVID-19: OR 3.13, 95% CI 2.22–4.01; influenza: OR 1.58, 95% CI 0.95–2.64).

### 3.2. Factors Associated with and Reasons for Shifting Attitudes in the 2023/2024 Seasonal Vaccination Campaign

**Figure 2:**
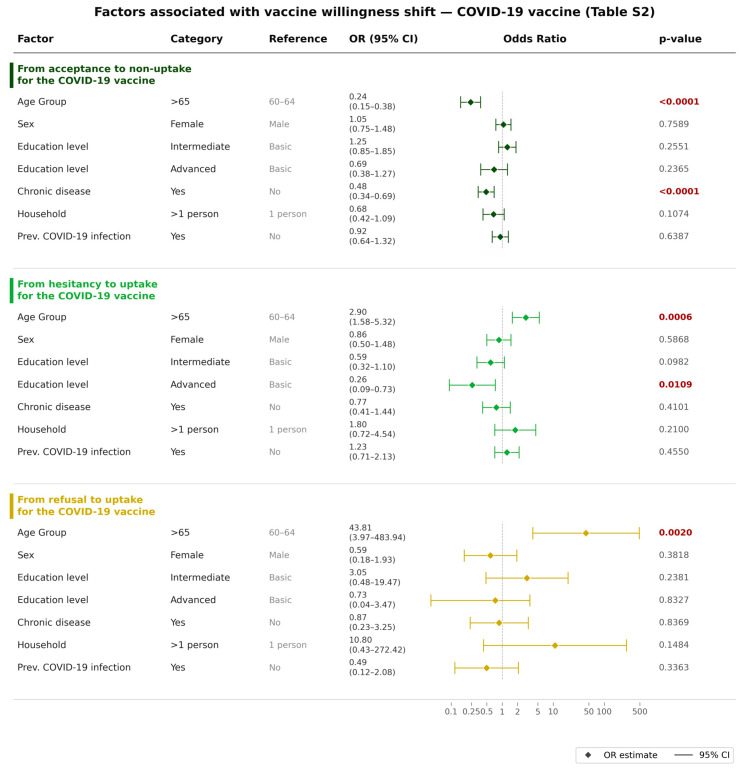
Representation of odds ratio values, and corresponding 95% confidence intervals of each factor, associated with shifting of attitudes toward the COVID-19 vaccine among individuals aged 60 years or older residing in mainland Portugal.

**Figure 3:**
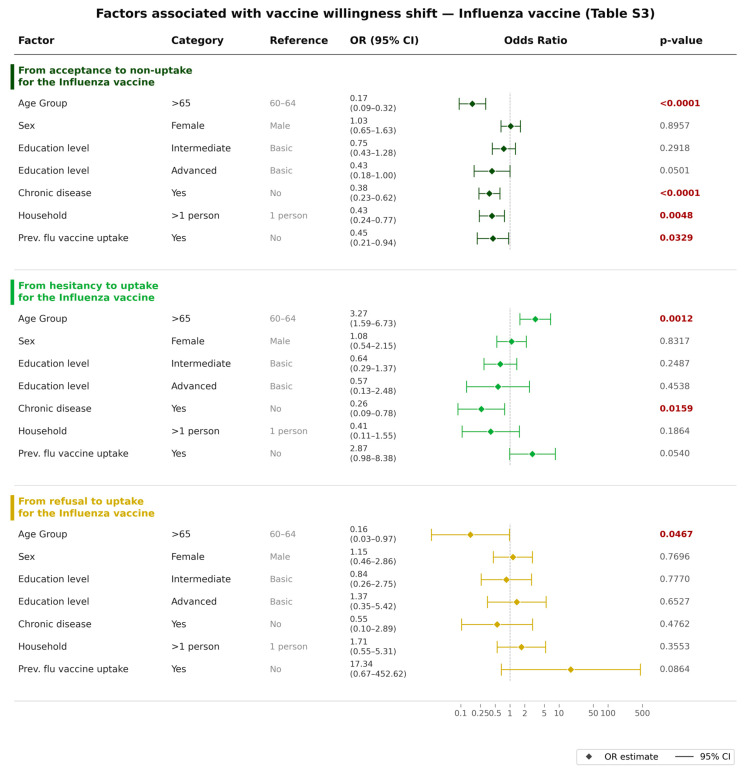
Representation of odds ratio values, and corresponding 95% confidence intervals of each factor, associated with shifting of attitudes toward the influenza vaccine among individuals aged 60 years or older residing in mainland Portugal.

Factors for shifting attitudes about the influenza and COVID-19 vaccines are presented in Figure 2 and Figure 3 and detailed in the Supplementary Materials (Table S2 and Table S3, respectively). The reported reasons for uptake and non-uptake decisions are also detailed in the Supplementary Materials (Table S4 and Table S5, respectively).

#### 3.2.1. From Acceptance to Non-Uptake

Among individuals who initially reported acceptance of COVID-19 vaccination, being aged 65 years or older (OR 0.24, 95% CI 0.15–0.38) and having a chronic disease (OR 0.48, 95% CI 0.34–0.69) were found to be factors associated with shifting, and, therefore, associated with uptake.

The leading self-reported reasons for non-uptake in this group included “lack of trust in the vaccine’s safety” (n = 155, 72.43%), “previous experience with side effects” (n = 26, 12.18%), and “mistrust in the vaccine’s effectiveness” (n = 14, 6.34%).

As for the influenza vaccine, among individuals who initially reported acceptance, being aged 65 years or older (OR 0.17, 95% CI 0.09–0.32), having a chronic disease (OR 0.38, 95% CI 0.23–0.62), living in a household with more than one person (OR 0.43, 95% CI 0.24–0.77), and having a history of prior influenza vaccination (OR 0.45, 95% CI 0.22–0.94) were found to be factors associated with shifting, and, therefore, associated with uptake.

The leading self-reported reasons for non-uptake in this group included “lack of trust in the vaccine’s safety” (n = 70, 69.41%), “mistrust in the vaccine’s effectiveness” (n = 19, 18.63%), and “previous experience with side effects” (n = 5, 4.87%).

#### 3.2.2. From Hesitancy to Uptake or Non-Uptake

Among individuals who reported hesitancy toward COVID-19 vaccination, being aged 65 years or older (OR 2.90, 95% CI 1.58–5.32) was associated with uptake, while having a higher education level (OR 0.26, 95% CI 0.09–0.73) was associated with non-uptake.

The leading self-reported reasons for uptake in this group included “self-initiative” (n = 59, 49.93%), “physician’s advice” (n = 37, 31.43%), and “risk awareness” (n = 30, 24.83%).

The leading self-reported reasons for non-uptake in this group included “low perceived susceptibility” (n = 102, 66.24%), “previous experience with side effects” (n = 21, 13.52%), and “vaccine fatigue” (n = 18, 11.61%).

As for the influenza vaccine, among individuals who reported hesitancy, being aged 65 years or older (OR 3.28, 95% CI 1.60–6.73) was associated with uptake, while having a chronic disease (OR 0.26, 95% CI 0.09–0.78) was associated with non-uptake.

The leading self-reported reasons for uptake in this group included “self-initiative” (n = 53, 37.87%), “risk awareness” (n = 48, 34.79%), “physician’s advice” (n = 47, 34.04%), and “being invited to vaccinate via SMS” (n = 17, 12.49%).

The leading self-reported reasons for non-uptake in this group included “low perceived susceptibility” (n = 44, 71.80%), “previous experience with side effects” (n = 8, 13.38%), “vaccine fatigue” (n = 18, 11.61%), and “lack of personal availability” (n = 6, 9.40%).

#### 3.2.3. From Refusal to Uptake

Among individuals who initially reported refusal for the COVID-19 vaccine, being aged 65 years or older (OR 43.815, 95% CI 3.967–483.938) was associated with shifting toward uptake; however, given the small number of participants in the refusal subgroup and the wide confidence interval, this estimate should be interpreted with considerable caution and regarded as exploratory.

The leading self-reported reasons for uptake in this group included “self-initiative” (n = 17, 62.30%), “physician’s advice” (n = 7, 24.59%), and “pharmacist’s advice” (n = 3, 11.49%).

Regarding the influenza vaccine, being aged 65 years or older (OR 0.156, 95% CI 0.025–0.973) was associated with not shifting from refusal to uptake, and, therefore, associated with non-uptake.

The leading self-reported reasons for uptake in this group included “self-initiative” (n = 14, 49.30%), “risk awareness” (n = 11, 37.71%), “physician’s advice” (n = 7, 24.58%), and “being invited to vaccinate via SMS” (n = 3, 10.75%).

## 4. Discussion

This study shows that attitudes toward COVID-19 and influenza vaccines can vary in all directions over a short period, such as the duration of a seasonal vaccination campaign. Understanding the factors and motivations that drive these rapid changes is, therefore, essential for designing timely, targeted, and effective public health strategies capable of sustaining vaccine confidence and improving uptake.

Although strongly associated, vaccine acceptance does not necessarily imply uptake. This highlights the need to sustain efforts to promote vaccination across all groups (including those most receptive to vaccination), and the importance of monitoring hesitancy beyond coverage. Similarly, refusal does not always result in non-uptake, challenging the existing literature that assumes refusal to be an immutable behavior.

Among those reporting acceptance, the decision for non-uptake is primarily driven by confidence factors. For the hesitant group, the main factors relate to complacency.

Regarding those who report refusal, the decision may be related to all three domains: complacency, confidence, and convenience. These findings are reported for their descriptive value in characterizing a subgroup that remains largely understudied in the literature; however, given the very small sample size of this group, the results lack the statistical power required to produce robust, generalizable evidence and must, therefore, be interpreted with considerable caution. Notably, the small size of this subgroup is itself consistent with the generally low prevalence of explicit vaccine refusal—as opposed to mere hesitancy—reported in Portugal, suggesting that the study sample may reasonably reflect the actual dimension of this group in the population.

Regardless, increasing convenience may also improve coverage by raising the likelihood of hesitant individuals encountering an opportunity to vaccinate, especially those who cite “lack of personal availability” as the reason for non-uptake.

Hesitancy towards the COVID-19 vaccine was higher than towards the influenza vaccine and appears to have increased compared to previous seasons. A 2021 study using the same measurement instrument as the present study reported an acceptance rate of 79.2% across all age groups in Portugal [11]. This trend is further corroborated by a continuous decline in seasonal vaccination coverage among individuals aged 60 years and above: 56.14% in 2023/2024 [12], 45.34% in 2024/2025 [13], and 38.6% in 2025/2026 [14]. However, direct comparisons over time should be interpreted cautiously because of differences in target population, campaign context, epidemiological circumstances, and timing of data collection. Individuals who presented hesitancy at the start of the campaign made different decisions regarding the two vaccines—more than half opted for uptake of the influenza vaccine, unlike what was observed for the COVID-19 vaccine.

Among those who reported acceptance, lack of trust in the vaccine’s safety was the most reported reason for non-uptake for both vaccines. In the case of the COVID-19 vaccine, having experienced side effects was the second-most-cited reason; for the influenza vaccine, mistrust in the vaccine’s effectiveness was more influential. The hesitant group reported a low perception of risk but also fear of the side effects for non-uptake. Vaccine fatigue is relevant within this group, which was specific to the COVID-19 vaccine. For both vaccines, a healthcare professional’s recommendation was reported as a reason for uptake among individuals who initially reported refusal. These findings underscore the need to maintain efforts promoting vaccination across the population regardless of reported willingness, but also the importance of tailoring such efforts according to each group’s specific behaviors.

Being aged 65 or older was associated with vaccine uptake across nearly all groups, whether through sustained acceptance or shifting from hesitancy or refusal. This aligns with the existing literature that links aging with reduced vaccine hesitancy. Those who reported refusal for the influenza vaccine were an exception, where being 65 or older was associated with non-uptake. This behavior, not observed for the COVID-19 vaccination, may stem from the low perceived risk of influenza among older adults in Portugal, as previously described [6]. Alternatively, these results may reflect the lower volatility of decision-making in the population 65 years old and above regarding the influenza vaccine. As this is a vaccine with which they have been familiar for years, attitudes toward it may be more established compared to the COVID-19 vaccination or when compared to individuals aged 60–64, who were eligible for free-of-charge influenza vaccination for the first time during this campaign.

Having one or more chronic diseases was associated with sustained vaccine acceptance throughout the campaign for both vaccines, reflecting perceived vulnerability and risk of infection and/or severe illness. However, it was also associated with non-uptake among hesitant individuals for the influenza vaccine, which may result from the greater weight given to fear of side effects rather than to the perceived severity of the disease in an undecided group. This interpretation is supported by the most reported reasons for non-uptake in this group—low perceived susceptibility and fear of side effects. Since, for both the hesitant and the refusal groups, physician recommendation and perceived risk were cited as reasons for uptake, it seems that the physician’s role is in advising every patient of the benefits of uptake and providing reliable and accessible information about vaccine side effects throughout the campaign. Furthermore, receiving an SMS invitation was reported as a reason for uptake of vaccination in these groups.

For the influenza vaccine, not living alone strengthened the decision to vaccinate, reflecting a perception of vaccination as necessary for others’ protection; this argument could be useful in interventions aimed at promoting vaccination.

Higher education level was associated with non-uptake in the hesitant group for the COVID-19 vaccine, suggesting the existence of specifically negative attitudes toward COVID-19 vaccination (particularly when compared to this group’s sustained acceptance of the influenza vaccine). This issue warrants more targeted and in-depth research, particularly considering the main reasons reported by hesitant individuals for non-uptake, such as low risk perception, fear of side effects, and vaccine fatigue.

This study has limitations, including the need for further validation of the willingness categorization tool, although its performance was assessed in the present dataset. The small number of participants in the refusal subgroup limited the precision of some estimates, which should, therefore, be interpreted as exploratory. Attrition analysis was limited to baseline characteristics, as follow-up vaccination outcomes were unavailable for participants lost to follow-up, and residual attrition bias cannot be excluded. In addition, formal ethical approval was issued after data collection had taken place, although the protocol had been submitted for ethical assessment in advance, and safeguards regarding voluntary participation, oral informed consent, data protection, and anonymized data access were implemented. The use of self-reporting to determine vaccination status and chronic disease presence may also be a limitation; however, this is considered unlikely, given the similarity between the proportion of vaccinated individuals in the sample and the actual coverage achieved during this seasonal vaccination campaign. While the potential for bias in measuring vaccination intention cannot be completely ruled out, it was mitigated through analysis of the validity of the instrument used.

## 5. Conclusions

Vaccine hesitancy remains an important public health concern in the Portuguese population and appears to differ between COVID-19 and influenza vaccination. Although hesitancy and refusal are grouped in the WHO’s definition, this study shows that hesitant individuals present different characteristics from those who outright refuse vaccination, highlighting the importance of distinguishing between these groups and tailoring interventions accordingly.

Understanding these behaviors is crucial for designing effective strategies to mitigate hesitancy, as they directly impact the decision to uptake/non-uptake during a campaign. Such understanding implies the need to measure and monitor hesitancy comprehensively and beyond coverage.

The study also demonstrates that individuals who report either acceptance or refusal can ultimately decide contrary to their initial stance during a campaign. Among those reporting acceptance, non-uptake is primarily driven by confidence-related factors. Among those reporting hesitancy, it is mainly due to complacency. Among those reporting refusal, the decision is influenced by all three domains—complacency, confidence, and convenience.

These findings highlight the need for tailored communication and intervention strategies throughout a vaccination campaign for all groups. Addressing the specific concerns and behavioral patterns of each group is essential to improve vaccination uptake and optimize public health outcomes. Future strategies should integrate behavioral insights to effectively address dynamic vaccination decision-making processes.

## Figures and Tables

**Table 1:** Distribution of the population, 60 years old or over, by reported uptake or non-uptake for the COVID-19 vaccine and for the influenza vaccine within each category of willingness in the 2023/2024 campaign.

Vaccine	Willingness	Uptake	Non-Uptake
COVID-19	All categories	62.67% (n = 750)	37.33% (n = 447)
COVID-19	Refusal 8.84% (n = 106)	25.67% (n = 27)	74.33% (n = 79)
COVID-19	Hesitancy 22.84% (n = 273)	43.50% (n = 119)	56.50% (n = 154)
COVID-19	Acceptance 68.32% (n = 818)	73.86% (n = 604)	26.14% (n = 214)
Influenza	All categories	78.58% (n = 941)	21.42% (n = 256)
Influenza	Refusal 10.32% (n = 124)	23.18% (n = 29)	76.82% (n = 95)
Influenza	Hesitancy 16.72% (n = 200)	69.40% (n = 139)	30.60% (n = 61)
Influenza	Acceptance 72.96% (n = 874)	88.52% (n = 773)	11.48% (n = 100)

## Data Availability

The data presented in this study is available upon request from the corresponding author.

## Supplementary Materials

The following supporting information can be downloaded at: https://www.mdpi.com/article/10.3390/vaccines14070555/s1, Table S1: Characteristics of the population within each category of willingness to vaccinate for COVID-19 and influenza (refusal, hesitancy, and acceptance) in the 2023/2024 campaign; Table S2: Association of factors with shifting from each willingness category to uptake/non-uptake—odds ratio values (OR) and corresponding 95% confidence intervals (CI95) and *p*-value for each category of the tested factors for the COVID-19 vaccine; Table S3: Association of factors with shifting from each willingness category to uptake/non-uptake—odds ratio values (OR) and corresponding 95% confidence intervals (CI95) and *p*-value for each category of the tested factors for the influenza vaccine; Table S4: Reported reasons (n) (%) for shifting to uptake or non-uptake from each willingness category for the COVID-19 vaccine among individuals aged 60 years or older residing in mainland Portugal; Table S5: Reported reasons (n) (%) for shifting to uptake or non-uptake from each willingness category for the influenza vaccine among individuals aged 60 years or older residing in mainland Portugal.

## Abbreviations

The following abbreviations are used in this manuscript:

CI	Confidence interval
OR	Odds ratio
SMS	Short message service
WHO	World Health Organization
