# Supplementary material for: Shifting Attitudes from Willingness to Uptake in COVID-19 and Influenza Vaccination—Associated Factors and Reported Reasons
Source: Vaccines (Basel). 2026 Jun 25;14(7):555. doi: 10.3390/vaccines14070555 (PMC13431380; doi:10.3390/vaccines14070555)
Supplement: Supplementary file 1 [file vaccines-14-00555-s001.zip › vaccines-4341820-supplementary.pdf]

vaccines

MDPI

Supplementary material S1

Shifting Attitudes from Willingness to Uptake in COVID-19 and Influenza Vaccination - associated factors and reported reasons

Table S1. Characteristics of the population within each category of willingness to vaccinate for COVID-19 and Influenza (refusal, hesitancy, and acceptance), in the 2023/24 campaign.

			Willingness n (%)		
**Vaccine**	**Factor**	**Category**	**Refusal**	**Hesitancy**	**Acceptance**
COVID-19	Age Group	60-64	39 (36.60%)	99 (36.07%)	125 (15.28%)
		>65	67 (63.40%)	175 (63.93%)	693 (84.72%)
	Sex	Male	49 (46.54%)	135 (49.46%)	413 (50.47%)
		Female	57 (53.46%)	138 (50.54%)	405 (49.53%)
	Education level	Basic	68 (64.31%)	142 (52.98%)	471 58.79%)
		Intermediate	27 (26.04%)	95 (35.58%)	239 (29.79%)
		Advanced	10 (9.64%)	31 (11.44%)	91 (11.42%)
	Chronic disease	No	72 (67.64%)	194 (71.06%)	393 (48.03%)
		Yes	34 (32.36%)	79 (28.94%)	425 (51.97%)
	Household	1 person	19 (18.00%)	30 (10.86%)	110 (13.44%)
		>1 person	87 (82.00%)	244 (89.14%)	708 (86.56%)
	Previous COVID-19 infection	No	43 (42.88%)	132 (48.93%)	510 (62.47%)
		Yes	58 (57.12%)	138 (51.07%)	306 (37.53%)
Influenza	Age Group	60-64	95 (77.27%)	74 (36.91%)	94 (10.73%)
		>65	28 (22.73%)	126 (63.09%)	780 (89.27%)
	Sex	Male	54 (43.38%)	98 (48.75%)	447 (51.13%)
		Female	70 (56.62%)	103 (51.25%)	427 (48.87%)
	Education level	Basic	38 (30.86%)	101 (53.41%)	541 (62.80%)
		Intermediate	53 (43.34%)	75 (39.77%)	233 (27.00%)
		Advanced	32 (25.80%)	13 (6.82%)	88 (10.21%)
	Chronic disease	No	107 (86.40%)	157 (78.61%)	395 (45.22%)
		Yes	17 (13.60%)	43 (21.39%)	479 (54.78%)
	Household	1 person	27 (21.95%)	20 (10.09%)	112 (12.79%)
		>1 person	96 (78.05%)	180 (89.91%)	762 (87.21%)
	Previous Influenza vaccine uptake	No	101 (81.74%)	75 (37.27%)	22 (2.50%)
		Yes	23 (18.26%)	126 (62.73%)	852 (97.50%)

Table S2. Association of factors with shifting from each willingness category to uptake/non-uptake - odds ratio values (OR) and corresponding 95% confidence intervals (CI95) and p-value for each category of the tested factors, for the COVID-19 vaccine.

Shift	Factor	Category	Reference	OR	CI95	CI95	p-value
From acceptance to non-uptake for the COVID-19 vaccine	Age Group	>65	60-64	0.240	0.153	0.375	<.0001
	Sex	Female	Male	1.055	0.750	1.483	0.7589
	Education level	Intermediate	Basic	1.255	0.849	1.855	0.2551
		Advanced	Basic	0.695	0.380	1.270	0.2365
	Chronic disease	Yes	No	0.483	0.338	0.691	<.0001
	Household	>1 person	1 person	0.676	0.419	1.089	0.1074
	Previous COVID-19 infection	Yes	No	0.916	0.637	1.319	0.6387
From hesitancy to uptake for the COVID-19 vaccine	Age Group	>65	60-64	2.901	1.582	5.320	0.0006
	Sex	Female	Male	0.860	0.499	1.482	0.5868
	Education level	Intermediate	Basic	0.593	0.319	1.102	0.0982
		Advanced	Basic	0.259	0.092	0.733	0.0109
	Chronic disease	Yes	No	0.769	0.412	1.436	0.4101
	Household	>1 person	1 person	1.805	0.717	4.543	0.2100
	Previous COVID-19 infection	Yes	No	1.233	0.712	2.134	0.4550
From refusal to uptake for the COVID-19 vaccine	Age Group	>65	60-64	43.815	3.967	483.938	0.0020
	Sex	Female	Male	0.589	0.180	1.928	0.3818
	Education level	Intermediate	Basic	3.051	0.478	19.471	0.2381
		Advanced	Basic	0.730	0.040	3.474	0.8327
	Chronic disease	Yes	No	0.871	0.234	3.245	0.8369
	Household	>1 person	1 person	10.803	0.428	272.418	0.1484
	Previous COVID-19 infection	Yes	No	0.494	0.118	2.078	0.3363

Table S3. Association of factors with shifting from each willingness category to uptake/non-uptake - odds ratio values (OR) and corresponding 95% confidence intervals (CI95) and p-value for each category of the tested factors, for the Influenza vaccine.

Shift	Factor	Category	Reference	OR	CI95	CI95	p-value
From acceptance to non-uptake for the Influenza vaccine	Age Group	>65	60-64	0.171	0.092	0.319	<.0001
	Sex	Female	Male	1.031	0.652	1.630	0.8957
	Education level	Intermediate	Basic	0.747	0.434	1.285	0.2918
		Advanced	Basic	0.430	0.185	1.001	0.0501
	Chronic disease	Yes	No	0.379	0.232	0.621	0.0001
	Household	>1 person	1 person	0.428	0.237	0.772	0.0048
	Previous Influenza vaccine uptake	Yes	No	0.449	0.215	0.937	0.0329
From hesitancy to uptake for the Influenza vaccine	Age Group	>65	60-64	3.275	1.595	6.727	0.0012
	Sex	Female	Male	1.078	0.540	2.151	0.8317
	Education level	Intermediate	Basic	0.636	0.294	1.373	0.2487
		Advanced	Basic	0.570	0.131	2.482	0.4538
	Chronic disease	Yes	No	0.260	0.087	0.777	0.0159
	Household	>1 person	1 person	0.405	0.106	1.548	0.1864
	Previous Influenza vaccine uptake	Yes	No	2.868	0.982	8.380	0.0540
From refusal to uptake for the Influenza vaccine	Age Group	>65	60-64	0.156	0.025	0.973	0.0467
	Sex	Female	Male	1.147	0.459	2.864	0.7696
	Education level	Intermediate	Basic	0.843	0.259	2.746	0.7770
		Advanced	Basic	1.371	0.347	5.419	0.6527
	Chronic disease	Yes	No	0.545	0.103	2.892	0.4762
	Household	>1 person	1 person	1.708	0.549	5.311	0.3553
	Previous Influenza vaccine uptake	Yes	No	17.344	0.665	452.617	0.0864

Table S4. Reported reasons (n) (%) for shifting to uptake or non-uptake from each willingness category for the COVID-19 vaccine, among individuals aged 60 years or older residing in mainland Portugal.

Shift	Reasons for the COVID-19 vaccine	N	%
From acceptance to non-uptake for the COVID-19 vaccine	Previous experience with side effects	26	12.18
	Lack of trust in the vaccine's safety	155	72.43
	Mistrust in the vaccine's effectiveness	14	6.34
	Not having received an invitation via phone call	10	4.79
	Lack of personal availability	10	4.77
	The vaccine was not available	8	3.95
	Perceived low susceptibility	2	0.94
	Vaccine fatigue	2	0.73
	Other	0	0
From refusal to uptake for the COVID-19 vaccine	Risk awareness	2	8.98
	Physician's advice	7	24.59
	Pharmacist's advice	3	11.49
	Work place initiative	0	0
	Self-initiative	17	62.30
	Was invited to vaccinate via SMS	2	5.74
From hesitancy to uptake for the COVID-19 vaccine	Risk awareness	30	24.83
	Physician's advice	37	31.43
	Pharmacist's advice	2	1,68
	Work place initiative	0	0
	Self-initiative	59	49.93
	Was invited to vaccinate via SMS	10	8.04
From hesitancy to non-uptake for the COVID-19 vaccine	Previous experience with side effects	21	13.52
	Lack of trust in the vaccine's safety	3	2.15
	Mistrust in the vaccine's effectiveness	0	0
	Not having received an invitation via phone call	2	1.01
	Lack of personal availability	10	6.61
	The vaccine was not available	0	0
	Perceived low susceptibility	102	66.24
	Vaccine fatigue	18	11.61
	Other	13	8.47

Table S5. Reported reasons (n) (%) for shifting to uptake or non-uptake from each willingness category for the Influenza vaccine, among individuals aged 60 years or older residing in mainland Portugal.

Shift	Reasons for the Influenza vaccine	N	%
From acceptance to non-uptake for the Influenza vaccine	Previous experience with side effects	5	4.87
	Lack of trust in the vaccine's safety	70	69.41
	Mistrust in the vaccine's effectiveness	19	18.63
	Not having received an invitation via phone call	3	3.11
	Lack of personal availability	2	2.43
	The vaccine was not available	2	1.56
	Perceived low susceptibility	2	1.56
	Vaccine fatigue	0	0
	Other	0	0
From refusal to uptake for the Influenza vaccine	Risk awareness	11	37.71
	Physician's advice	7	24.58
	Pharmacist's advice	0	0
	Workplace initiative	0	0
	Self-initiative	14	49.30
	Was invited to vaccinate via SMS	3	10.75
From hesitancy to uptake for the Influenza vaccine	Risk awareness	48	34.79
	Physician's advice	47	34.04
	Pharmacist's advice	0	0
	Workplace initiative	0	0
	Self-initiative	53	37.87
	Was invited to vaccinate via SMS	17	12.49
From hesitancy to non-uptake for the Influenza vaccine	Previous experience with side effects	8	13.38
	Lack of trust in the vaccine's safety	0	0
	Mistrust in the vaccine's effectiveness	0	0
	Not having received an invitation via phone call	0	0
	Lack of personal availability	6	9.40
	The vaccine was not available	0	0
	Perceived low susceptibility	44	71.80
	Vaccine fatigue	0	0
	Other	3	5.42
